# Oral Manifestations in SARS-CoV-2 Positive Patients: A Systematic Review

**DOI:** 10.3390/jcm11082202

**Published:** 2022-04-14

**Authors:** Kacper Nijakowski, Sylvia Wyzga, Nisha Singh, Filip Podgórski, Anna Surdacka

**Affiliations:** 1 Department of Conservative Dentistry and Endodontics, Poznan University of Medical Sciences, 60-812 Poznan, Poland; annasurd@ump.edu.pl; 2 Student’s Scientific Group in Department of Conservative Dentistry and Endodontics, Poznan University of Medical Sciences, 60-812 Poznan, Poland; sylviawyzga95@gmail.com (S.W.); singhnisha6070@gmail.com (N.S.); pod.filip@outlook.com (F.P.)

**Keywords:** COVID-19, SARS-CoV-2, oral manifestation, oral lesion, oral health

## Abstract

The COVID-19 pandemic has severely affected the human population by revealing many health problems, including within the oral cavity. This systematic review was designed to answer the question “Is there a relationship between oral manifestations and SARS-CoV-2 infection?”. Following the inclusion and exclusion criteria, twenty-seven studies were included (according to PRISMA statement guidelines). Based on the meta-analysis, nearly two-thirds of the SARS-CoV-2 positive patients reported oral symptoms, in particular taste alterations, xerostomia and ulcerations (54.73% [95% CI: 46.28–63.04%], 37.58% [95% CI: 26.35–49.53%], and 21.43% [95% CI: 13.17–31.06%], respectively). In conclusion, despite the conducted systematic review, the increased prevalence of oral manifestations in SARS-CoV-2 infection cannot be clearly established due to the possible association of other factors, e.g., individual or environmental factors.

## 1. Introduction

Multiple cases of pneumonia of unknown etiology were reported in medical facilities in Wuhan city at the end of 2019. Researchers confirmed that the acute respiratory infection was caused by a novel coronavirus. On 7 January 2020 the Chinese Centre for Disease Control and Prevention (CCDC) identified the causative agent and named its Severe Acute Respiratory Syndrome Coronavirus 2 (SARS-CoV-2) [1]. The disease was named Coronavirus disease 2019 (COVID-19) by Director-General of The World Health Organization (WHO). The disease quickly spread among many parts of the world [2]. WHO declared the outbreak of a global pandemic of COVID-19 on 11 March 2020 [3].

SARS-CoV-2 is the seventh known coronavirus able to infect humans and is capable of causing severe infection alongside SARS-CoV and MERS-CoV (Middle East Respiratory Syndrome Coronavirus) [4]. Transmission of the virus is based on direct contact of respiratory droplets with mucous membrane [5]. Studies show that aerosol and airborne methods of transmission of SARS-CoV-2 are a crucial pathway of disease spreading [6,7]. SARS-CoV-2 RNA (ribonucleic acid) was present in air samples from hospitals in Wuhan city [8]. It is also possible to become infected through contact with contaminated objects [9].

The asymptomatic incubation period of SARS-CoV-2 was estimated to be between 2.2 and 12.5 days [10]. The most common systemic symptoms of COVID-19 are fever, cough, diarrhea and fatigue [11,12]. However, some of the patients with a severe course of disease have developed fatal complications such as organ failure, septic shock, pulmonary edema, severe pneumonia, and Acute Respiratory Distress Syndrome (ARDS) [13]. Antiviral agents such as lopinavir or remdesivir are helpful in suppressing the progression of COVID-19. Furthermore, an improvement in survivability was found in patients treated with plasma and hyperimmune immunoglobulins [14]. Social isolation, washing and disinfecting hands, and wearing a mask in public proved effective in avoiding disease and reducing transmission of the virus [9].

The outbreak of the pandemic also made a huge impact on human mental health. Each individual dealt with various stressors such as fear of death or health complications, worry about one’s financial status related to the massive loss of workplaces due to restrictions, and social isolation [15,16]. People also started to experience more negative emotions such as loneliness, unhappiness, depression, anger, emotional exhaustion and more [17,18,19]. Despite all of these changes people adapted well to the situation and seemed to find a way of fulfilling their social and emotional needs [20].

The COVID-19 pandemic had its challenging outcome on dental care. The shutdown of academic institutions had its impact on the quality of education of dental students and subsequently led to a growth in the number of patients in need of urgent dental care [21,22,23]. The transmission of SARS-CoV-2 through aerosol is a main problem during dental treatment, associating it with a high risk of infection. In order to prevent the transmission of the virus, guidelines recommend wearing an N95 mask, protective clothing, eye protector, latex gloves, surgical cap and shoe covers [24]. The increasing number of COVID-19 survivors forced dentists to come out with solutions and find ways to provide patients in need of dental care with a multi-professional approach [25]. The factors discussed above regarding psychological isolation and limited access to dental care may have influenced the occurrence and development of changes in the oral mucosa in the course of SARS-CoV-2 infection.

Our systematic review was designed in order to answer the question: “Is there a relationship between oral manifestations and SARS-CoV-2 infection?”; formulated according to PICO (“Population”, “Intervention”, “Comparison” and “Outcome”).

## 2. Materials and Methods

### 2.1. Search Strategy and Data Extraction

A systematic review was conducted up to 5th March 2022, according to the Preferred Reporting Items for Systematic Reviews and Meta-Analyses (PRISMA) statement guidelines [26], using the databases PubMed, Scopus and Web of Science. The search formulas included:

- For PubMed: COVID AND (oral AND ((manifestation*) OR (lesion*)))
- For Scopus: TITLE-ABS-KEY(COVID* AND (oral AND (manifestation* OR lesion*)))
- For Web of Science: TS = (COVID* AND (oral AND (manifestation* OR lesion*))).

Records were screened by the title, abstract and full text by two independent investigators. Studies included in this review matched all the predefined criteria according to PICOS (“Population”, “Intervention”, “Comparison”, “Outcomes”, and “Study design”), as shown in Table 1. A detailed search flowchart is presented in the Results section.

The study protocol was registered in the International prospective register of systematic reviews PROSPERO (CRD42022315223). The results of the meta-analysis are presented in forest plots using MedCalc Statistical Software version 19.5.3 (MedCalc Software Ltd., Ostend, Belgium).

### 2.2. Quality Assessment and Critical Appraisal for the Systematic Review of Included Studies

The risk of bias in each individual study was assessed according to the “Study Quality Assessment Tool” issued by the National Heart, Lung, and Blood Institute within the National Institute of Health [27]. These questionnaires were answered by two independent investigators, and any disagreements were resolved by a discussion between them.

**Figure 1:**
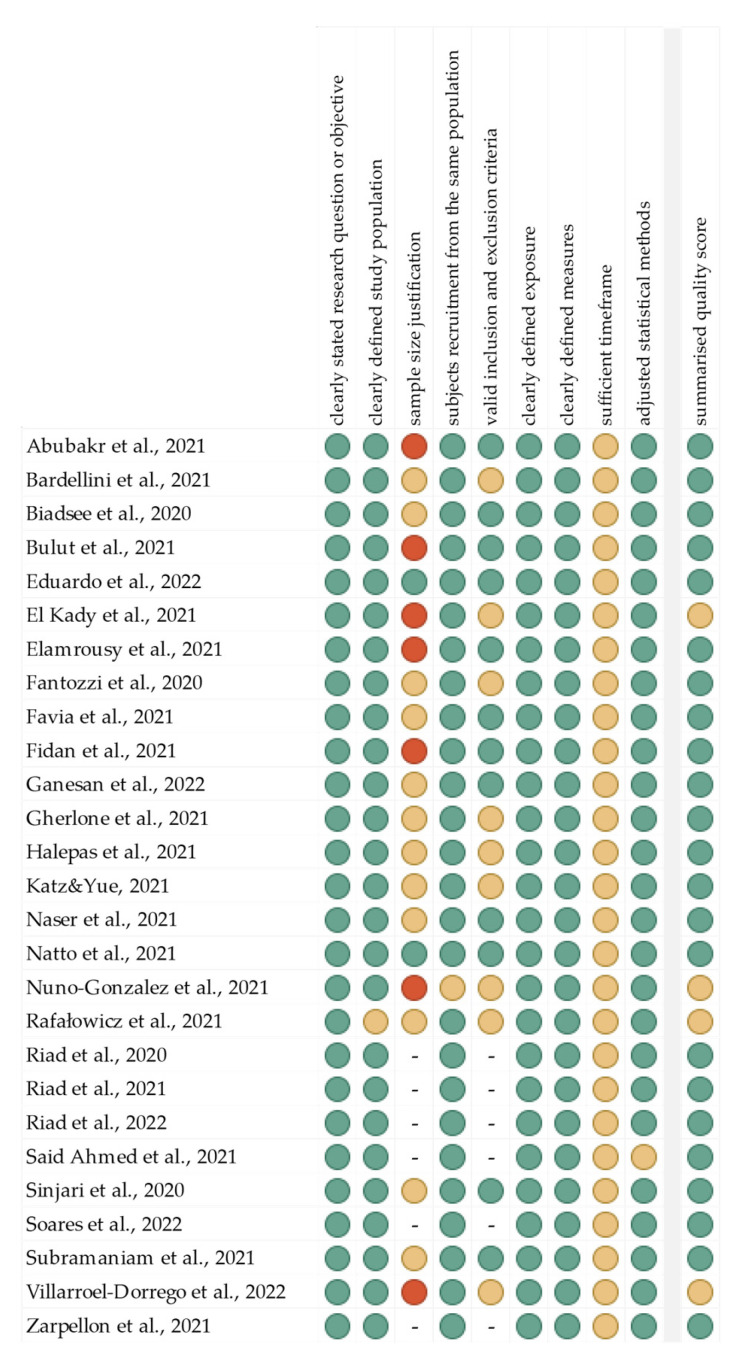
Quality assessment, including the main potential risk of bias. (Risk level: green—low; yellow—unspecified; red—high. Quality score: green—good; yellow—intermediate; red—poor).

The summarized quality assessment for every single study is reported in Figure 1. The most frequently encountered risks of bias were the absence of data regarding sample size justification (except two studies), and sufficient timeframe (all studies). Critical appraisal was summarized by adding up the points for each criterion of potential risk (points: 1—low, 0.5—unspecified, 0—high). A total of 23 studies (85.2%) were classified as having “good” quality (≥80% total score) and four (14.8%) as “intermediate” (≥60% total score).

The level of evidence was assessed using the classification of the Oxford Centre for Evidence-Based Medicine levels for diagnosis [28]. All of the included studies have the third or fourth level of evidence (in this 5-graded scale).

## 3. Results

**Figure 2:**
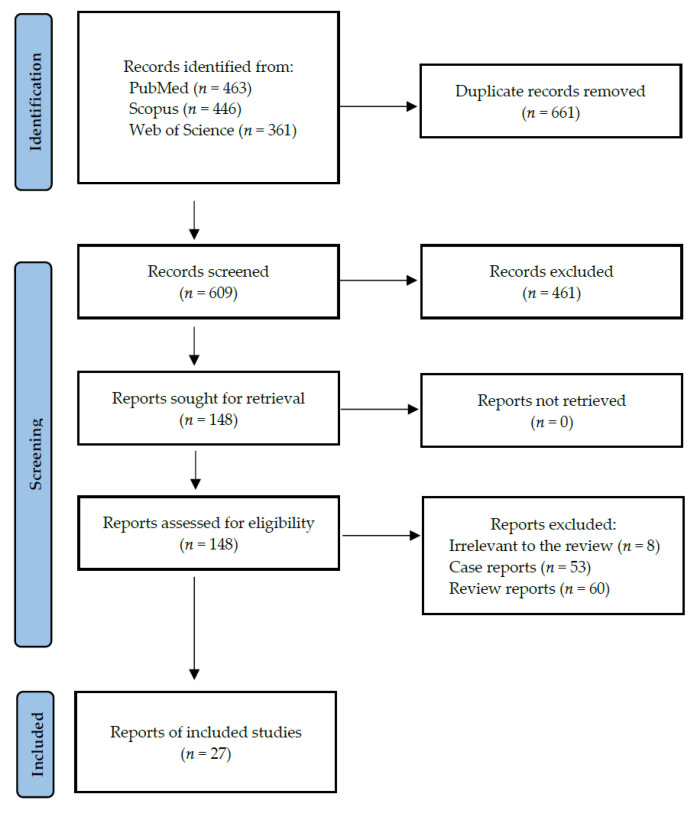
PRISMA flow diagram presenting search strategy.

Following the search criteria, our systematic review included twenty-seven studies, demonstrating data collected in twelve different countries from a total of 6722 participants with diagnosed SARS-CoV-2 infection (including 2476 females and 2990 males, and 1256 patients without reported gender). Figure 2 shows the detailed selection strategy of the articles. The inclusion and exclusion criteria are presented in Table 1 (in the Section 2).

From each eligible study included in the present systematic review, we collected data about its general characteristics, such as the year of publication and setting, involved participants (gender, age, co-morbidities), COVID-19 treatment and hospitalization, inclusion and exclusion criteria, general manifestations, and smoking status (Table 2). Table 3 presents the detailed characteristics considering oral manifestations, including investigation, type, location and frequency of oral lesions.

**Figure 3:**
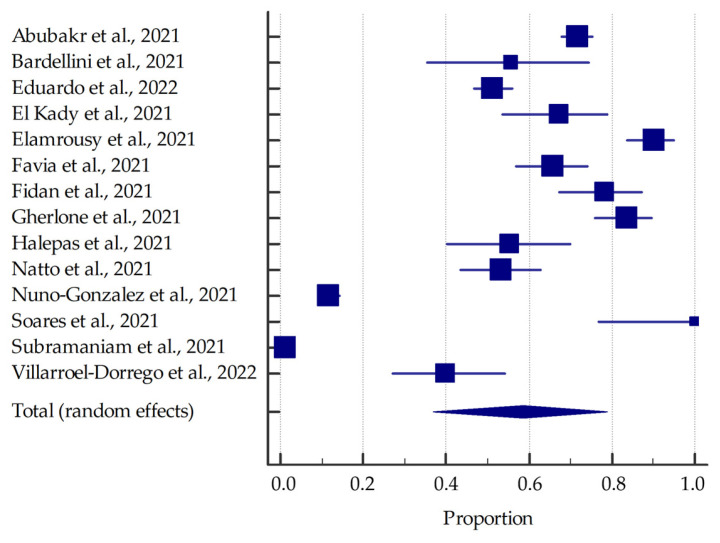
Forest plot presenting the summarised prevalence of oral manifestations among SARS-CoV-2 positive individuals.

**Figure 4:**
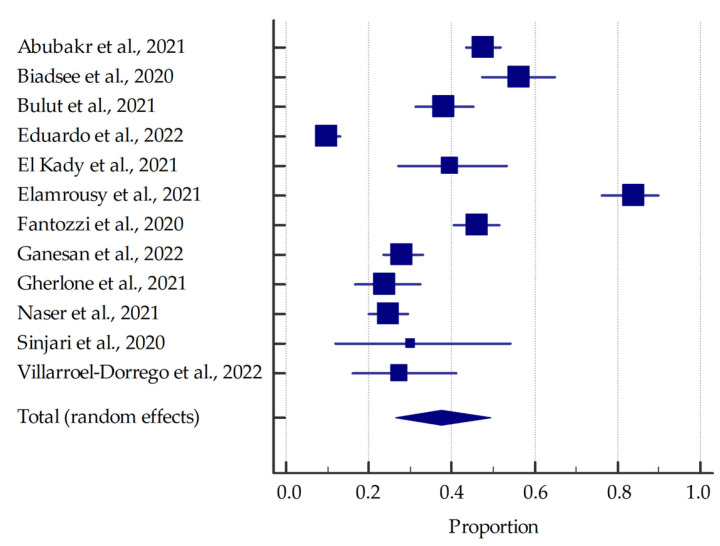
Forest plot presenting the summarized prevalence of xerostomia among SARS-CoV-2 positive individuals.

**Figure 5:**
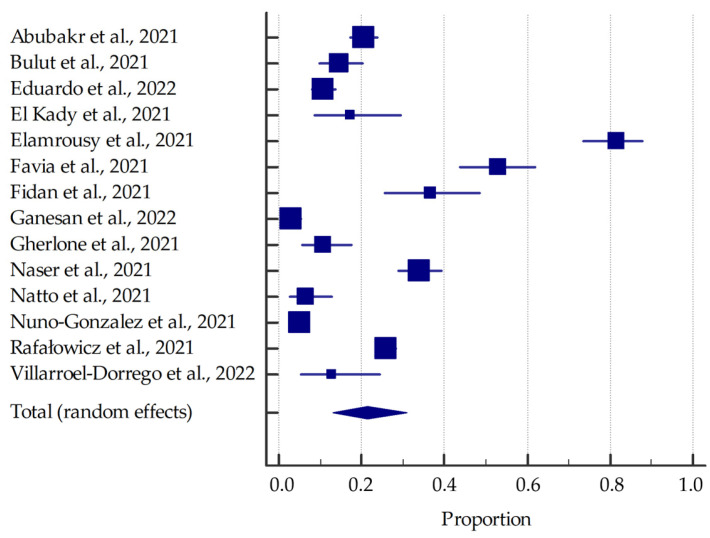
Forest plot presenting the summarized prevalence of ulcerations among SARS-CoV-2 positive individuals.

**Figure 6:**
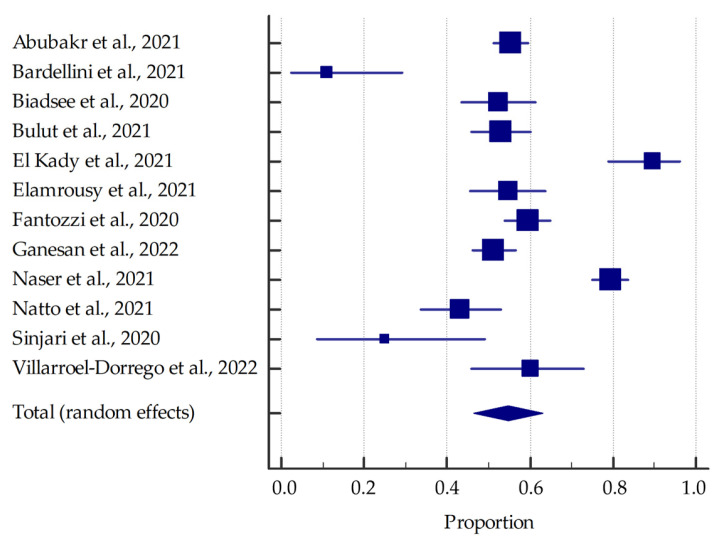
Forest plot presenting the summarized prevalence of taste alterations among SARS-CoV-2 positive individuals.

Most studies (involving at least 20 patients) were included in the meta-analysis, the results of which are presented in the forest plots (Figure 3, Figure 4, Figure 5 and Figure 6).

Based on the included studies reporting the frequency of oral manifestations in SARS-CoV-2 positive individuals, it was determined that the aggregate prevalence was approximately 58.75% [95% CI: 36.93–78.90%]. Additionally, in this group, the summarized prevalence of taste alterations, xerostomia and ulcerations were estimated to be around 54.73% [95% CI: 46.28–63.04%], 37.58% [95% CI: 26.35–49.53%], and 21.43% [95% CI: 13.17–31.06%], respectively.

## 4. Discussion

In our discussion, the literature reviewed in regards to oral manifestations of SARS-CoV-2 infection were divided into two subgroups: descriptive studies (surveys, case series) and analytical studies (observational: cross-sectional, prospective or retrospective).

### 4.1. Descriptive Studies

A study conducted by Abubakr et al. [29] surveyed Egyptian patients who presented with mild-moderate cases of COVID-19 and suffered from one or more oral manifestations due to the virus. As such, 47.6% of the participants experienced xerostomia, 23% suffered from oral or dental pain, 20.4% reported the appearance of ulcerations in the oral cavity with a significant occurrence in males when compared to females, 12% complained of pain in the jaw bones or joints, and 10.5% of patients felt they had halitosis. Moreover, 28.3% of COVID-19 patients simultaneously experienced two or three of these oral manifestations. This study also examined oral hygiene status among these infected patients. It confirmed that patients with poor oral hygiene had ulcerations and oral/dental pain more often than those with good oral hygiene. The most prevalent oral manifestation was xerostomia, and the least pervasive was halitosis.

Similarly, Biadsee et al. [31] examined a web-based questionnaire distributed to 128 patients with COVID-19 who were quarantining in designated hotels in Israel. The following oral manifestations reported are olfactory dysfunction, taste alterations, dry mouth, facial pain, and masticatory muscle pain. As such, eighty-six patients (67%) had olfactory dysfunction, with 19.5% of patients who felt it was heightened from day three to five of the infection. A total of sixty-seven patients (52%) experienced changes in their taste sensation, specifically toward spicy, salty, sour, and sweet foods, and women were impacted more than men. Furthermore, seventy-two patients reported dry mouth, strongly correlated to burning mouth and taste alterations. Facial pain was reported by eighteen patients (26%), and fifteen patients (11%) experienced masticatory muscle pain. Additionally, twenty patients described changes to their tongue sensation, nine patients reported the appearance of plaque on their tongue, and three patients reported oral bleeding. Lastly, oral hygiene did not contribute to these manifestations.

In a study by El Kady et al. [34], a pilot survey on Google Form was conducted on SARS-CoV-2 positive patients, asking the patients to report any oral manifestations listed in the questionnaire, and four categories were created: 1. Gustatory disorders (loss of salt sensation, loss of sweetness, and altered food taste); 2. Symptoms of salivary gland infection (dry mouth, difficulty swallowing, pain or swelling in the submandibular gland area, and pain or swelling in the parotid gland area); 3. Oral mucosal changes (oral ulcers, spots on mouth or lips, tongue redness, gingival bleeding, and burning sensation); 4. Category for patients with no symptoms related to the oral cavity and salivary gland. The results reported that 67.2% patients had at least one oral manifestation, with the highest prevalence symptom of dry mouth at 39.7%. Other symptoms of gustatory dysfunction were 34.5% loss of salt sensation, 29.3% loss of sweetness and 25.9% altered food taste. In this study, the highest percentage of occurrence of taste alterations among all the reports included in the meta-analysis was found. Salivary gland infection at 22.4% difficult swelling, 13.8% pain or swellings in the salivary gland or cheek, and 10.3% pain or swelling below the mandible. Oral mucosal changes were less prevalent. Loss of salt and sweetness were most associated (27.6%), and dry mouth and gustatory impairment symptoms were often associated (27.6%). The high prevalence of salivary gland related symptoms highlights the protective role and value of saliva against viruses.

Moreover, in a survey study by Bulut et al. [32], the possible effects of SARS-CoV-2 on the oral tissues were investigated. A total of 200 volunteers, who survived COVID-19, filled out a questionnaire after routine clinical and radiographic examinations, regarding their demographic characteristics, general health, oral habits and symptoms in the oral cavity. During the active period of SARS-CoV-2 infection the following observations of the oral cavity were recorded; taste loss (53%), halitosis (21%), oropharyngeal wound and pain (18%), pain in temporomandibular joint (17.5%), pain in the chewing muscles (16%), aphthous ulcer (14.5%), sensitivity and/or pain in teeth (12%), herpes labialis (8.5%), and burning in the tongue (7.5%). Xerostomia was observed in 38% of the patients during the active period, and of those, 27.6% continued to have xerostomia after. The issue with halitosis can be due to the wound and pain in the oropharynx region, infection of tonsils and the soft palate leading to the collection of bacteria and fungi.

In contrast, Riad et al. [49] conducted a case series on patients who sought care in their hospital for pain in the tongue and had a confirmed SARS-CoV-2 laboratory test. General symptoms experienced by these patients included only one oral manifestation, which is ageusia. The onset of tongue ulcers was after five days for 53.8% of patients, 26.9% after four days, and 7.7% after two days. The number of ulcers varied considerably among these individuals, and their size ranged from 1 to 5 mm. The location of these ulcers was predominately on the dorsum of the tongue, while a few patients had them on the ventral surface of the tongue.

In another study by Riad et al. [47], thirteen patients with oral mucositis were examined. These patients complained of generalized pain and soreness within the oral cavity, focused on the nonkeratinized mucosa. The onset of mucositis was recorded at 0–2 days after a positive PCR SARS-CoV-2 test was done, and the mean duration was 7–14 days. Intraoral examinations found depapillation of the tongue in all cases, sporadic erythema (53.8%) on the buccal mucosa (30.8%), palate (15.4%) and gingiva (7.7%). Significant associations of COVID-19 severity and duration of mucositis with pain, and the loss of sense of taste associated with mucositis durations was disclosed. These results support the role of oral mucosa in being the entryway for the virus. Patients with COVID-19 may easily present with mucositis due to cellular damage triggered by the virus, or as an opportunistic infection due to immune deregulation.

Furthermore, Riad et al. [48] reported eighteen patients who presented a new-onset halitosis during the course of COVID-19 infection. The issue was brought up due to an offensive oral malodor that precipitated notable psychosocial distress, in which the Halimeter Plus was used for quantitative assessment. Oral hygiene states were assessed with the Oral Health Assessment Tool finding that most of the patients had a fair level of oral hygiene, therefore suggesting that the epithelial alterations by SARS-CoV-2 on the dorsum of the tongue is the probable reason for the halitosis.

Interestingly, Said Ahmed et al. [50] present a series of fourteen cases that discuss the prevalence of maxillary mucormycosis osteomyelitis in post-COVID-19 patients. In this study, nine patients had diabetes before contracting COVID-19, and five patients did not, but both groups showed signs of hyperglycemia after their quarantine. This case report concluded that patients with diabetes are at a higher risk of maxillary mucormycosis osteomyelitis due to drugs used to treat COVID-19. It is suggested that these medications can cause patients to become immunocompromised, primarily due to their diabetic status.

Rafałowicz et al. [46] studied 1256 patients, and of those presented 6 cases of patients with the most common long COVID-19 oral cavity symptoms. Amidst these patients the following oral cavity changes were reported: 32% discolouration, ulcerations and hemorrhagic changes on the oral mucosa, 29.69% mycosis on the tongue, 25.79% aphthous-like lesion on hard palate, and 12.5% atrophic cheilitis.

Based on the included descriptive studies, xerostomia, olfactory dysfunction and changes in taste sensation were the most prevalent oral manifestation in SARS-CoV-2 positive patients. Halitosis, masticatory muscle pain, and burning sensation were the least prevalent and were rarely observed.

### 4.2. Analytical Studies

In an observational study by Sinjari et al. [51], patients hospitalized due to COVID-19 participated in a questionnaire to better understand the oral manifestation of these patients. Health status, oral hygiene habits, and symptoms in the oral cavity before and during the hospitalization/COVID-19 manifestation were collected. Additionally, questions to the Unit of Internal Medicine of the hospital, in regards to the clinical conditions of the patients, were collected. As such, 30% of patients reported xerostomia during hospitalization, 25% impaired taste, 15% burning sensation, and 20% difficulty in swallowing. Nuno-Gonzalez et al. [45] examined 666 patients with COVID-19 in a field hospital in Spain to determine the prevalence of oral and palmoplantar mucocutaneous lesions in infected individuals. The authors established the following results: 25.7% had oral manifestations, including 11.5% transient lingual papillitis, 6.6% aphthous stomatitis, 3.9% glossitis with patchy depapillation, and 3.9% with mucositis. Other common adjuncts to these oral manifestations are burning sensation, which was felt among 5.3% of patients, and taste disturbances.

Gherlone et al. [40] completed a retrospective and prospective cohort study that included COVID-19 patients admitted to San Raffaele University Hospital in Milan. The most frequent oral manifestations were salivary gland ectasia, dry mouth, TMJ abnormalities, and masticatory muscle weakness. In total, 38% of patients suffered from salivary gland ectasia, making it this study’s most common oral manifestation. The majority of cases were found among the male patients, individuals who had a severe case of COVID-19, and patients who received antibiotics during hospitalization. At follow-up evaluations, dry mouth was found within 30% of the patients. Diabetes mellitus and COPD are associated with dry mouth; therefore, these medical conditions significantly increase the likelihood of dry mouth within these individuals. Masticatory muscle weakness was found among 19% of patients, 17% suffered from dysgeusia, 14% anosmia, 7% TMJ abnormalities, 3% facial tingling, and 2% trigeminal neuralgia.

Moreover, Fantozzi et al. [36] conducted a retrospective study that included patients admitted to the emergency department in Italy and had a confirmed diagnosis of COVID-19. This study determined the prevalence of xerostomia and gustatory/olfactory dysfunction among these individuals. Olfactory alterations were the most severe, followed by dysgeusia and xerostomia. A total of 60% of patients reported taste alterations, 45.9% suffered from xerostomia, and 41.4% reported smell dysfunctions, and 70% of patients suggested that these symptoms occurred before their COVID-19 diagnosis.

In a study by Villarroel-Dorrego et al. [54], 55 hospitalized patients, of which 19 were admitted to the intensive care unit, were examined for oral lesions. A total of 40% of patients presented with at least one lesion or variation. Ulcers, both hemorrhagic and aphthous-like, were the most common, and it is believed this manifestation is due to the virus directly causing damage to the oral tissue. Secondary manifestations on the buccal mucosa are commonly seen, specifically candidiasis and recurrent herpetic infection. Effects on the loss of taste (60%), pain or burning in mouth (36.4%), and xerostomia (27.3%) were also reported.

Similarly, Eduardo et al. [33] conducted a retrospective study on COVID-19 patients in the intensive care unit, in which 51.3% presented oral lesions and/or saliva alterations. Majority of the lesions in this study are due to the intubation process by an orotracheal tube, causing traumatic injuries (such as intraoral and extra-oral ulcerations) and the medications used in treatment, such as anticoagulants and corticosteroid-induced immunosuppression. Petechial and bruising can be from anticoagulants, however it is believed that it can also be possibly linked with SARS-CoV-2′s role in the microvasculature of the oral cavity. Dryness of the oral cavity is reported frequently as well.

In contrast, Bardellini et al. [30] completed a retrospective study among children infected with COVID-19 at a Pediatric Hospital in Brescia, a city in Italy with the highest number of COVID-19 cases. The authors reported the following findings among the examined cohort: hyperaemic pharynx, oral pseudomembranous candidiasis, coated tongue, geographic tongue which developed while the individual had a high fever, and lastly, alterations in taste and loss of appetite. Some children also presented with cutaneous lesions such as non-itchy confluent flat papular lesions of the face and limb but did not have any oral manifestations. Hyperaemic pharynx was the more frequent oral manifestation presented among the patients. The authors have suggested no consistent oral manifestations associated with COVID-19 in children, but instead typically found these clinical presentations in influenza virus infection.

Furthermore, Halepas et al. [41] conducted a cross-sectional study of pediatric patients admitted at Morgan Stanley Children’s Hospital of New York-Presbyterian and tested positive for SARS-CoV-2. This study aimed to determine if multisystem inflammatory syndrome increased the incidence of oral manifestations among positive individuals. Findings of this study are: 48.9% of patients had red or swollen lips, and 10.6% had strawberry tongue. In conclusion, these clinical appearances were present alongside systemic conditions such as full-body rash and conjunctivitis.

In a study by Favia et al. [37], 123 patients were observed for oral lesions, in which 65.9% of the oral lesions took place in the early stage of COVID-19. The early stage is defined as lesions that “appeared together with the onset of general symptoms or within one week and always before the beginning of COVID-19-specific therapies”. The most frequent lesions were single and multiple ulcers (52.8%) with a likelihood of developing into a larger necrotic area. Blisters were identified in 15.4% cases and of those, about 10% were mentioned as early ulcerative lesions in the medical record and exam of the patients. Several kinds of candidiasis were identified in 28 patients.

Ganesan et al. [39] conducted an observational cross-sectional study in a facility dedicated to SARS-CoV-2 positive patients. The yielded results are: 51.2% of patients experienced an alteration in taste sensation followed by complete ageusia, 28% of patients had xerostomia, and 15.4% of patients had mucosal, hard tissue, or bony lesions. Erythematous macules were the most common soft tissue lesions observed, followed by non-specific solitary ulcers, atrophic glossitis, and candida-like lesions.

Furthermore, in a study by Elamrousy et al. [35], 124 patients were admitted to explore if oral lesions affect the tongue mainly due to the greater number of cells expressing angiotensin-converting enzyme 2 than in other oral tissues. A total of 90.3% of patients presented with oral manifestations, of which 62% were asymptomatic. Dry mouth was examined in about 84% of the patients. Those that were symptomatic described a painful or burning sensation. Multiple types of oral lesions were identified, however oral ulcers (mostly aphthous-like ulcers covered with pseudomembrane) counted for the highest amount in 92.8% of patients. The most common sites for oral ulcers were the lip (with hemorrhagic ulcers with crust), tongue, and labial mucosa. Overall, the tongue presented with the highest amount of oral lesions at 85.7%, such as ulcers, atrophy, or combination of lesions with *Candida* infection. Among all the included studies above, the highest prevalence of xerostomia and ulcerations were observed in this study.

A cross-sectional study was conducted by Natto et al. [44] to determine the prevalence of oral manifestations in SARS-CoV-2 positive patients. 109 patients were examined in a single medical center with the following results: 43.4% lost their taste, 7.3% had erythema/desquamated gingivitis and coated tongue, lastly, 6.4% had visible ulcers/blisters. Loss of taste was the most common oral manifestation, and patients reported that it persisted for ten days. Other symptoms appeared for seven days or less, and 79.3% appeared as a single symptom. The most common site for these oral diseases to occur was the dorsum of the tongue (72.4%), followed by vestibules (12.1%) and gingiva, lips, buccal mucosa (8.6%).

Fidan et al. [38] examined 74 SARS-CoV-2 positive patients at their clinic and included them in their observational study. The results suggested that aphthous-like ulcers were the most common oral manifestation in 27 patients. Other findings were also recognized, such as erythema, which occurred in 19 patients, and lichen planus among 12 patients. The most common site for these clinical appearances was on the tongue (*n* = 27), and other areas included the buccal mucosa (*n* = 20), gingiva (*n* = 11), and palate (*n* = 4). In a study by Naser et al. [43], 338 patients were observed for identifying the most common oral and maxillofacial lesions. The most common oral conditions were the presence of a white coat on the tongue, pain related to the oral cavity, multiple aphthous ulceration in the oral cavity, dryness of the oral cavity, white coat of the gingiva and cheek, and white coat of the palate.

Surprisingly, in a study by Subramaniam et al. [53], only nine patients out of 713 reported oral discomfort due to some form of oral lesions. After oral examination, these oral lesions were found to range from herpes simplex ulcers to angular cheilitis. The authors conclude that they support that oral manifestations could be secondary lesions resulting from the deterioration of systemic health, or due to treatments for COVID-19, or could be just coexisting conditions.

In contrast, in a study by Soares et al. [52], fourteen patients were studied who presented oral lesions due to COVID-19. The identified oral lesions were divided into two groups, and a combination of the two groups also occurred: 1. Lesions presenting as ecchymosis, purplish areas, and petechial. These alterations were most commonly observed in the palate and tongue; 2. Vesiculobullous lesions or ulcerations with ischemic aspects, occurring in any location of the oral mucosa, but mainly on the lips, buccal mucosa, and tongue. Furthermore, eight of the fourteen patients presented with lesions only located on the palate (57.1%), four patients with tongue lesions, and two patients (14.3%) with lesions on lip or palate (14.3%). Clinical features consisted of petechial, ecchymosis, reddish macules, chronic ulcers, vesiculobullous eruptions in the lip, palate and buccal mucosa. In other studies by these authors, clinically and histopathologically it is confirmed that ulcers associated with COVID-19 have a distinguishing appearance, where ischemic borders and central area with a fibrinous pseudomembrane is identifiable, and evolves in about 21–28 days.

A brief report presented by Katz and Yue [42] examined patients with COVID-19 in Florida that included outpatients and inpatients at different health centers across the state. The study determined if there was an increased odds ratio for COVID-19 patients with recurrent aphthous stomatitis. The hospital population served as the control group, and the prevalence of recurrent aphthous stomatitis was 0.148% compared with 0.64% in the COVID-19 group. The odds ratio was adjusted based on demographics and comorbidities. The authors suggest a strong association between COVID-19 and recurrent aphthous stomatitis; however, more data is required.

Interestingly, Zarpellon et al. [55] conducted a post-mortem study on twenty-six deceased patients due to COVID-19. The autopsies included a thorough examination of the hard palate, tongue, jugal and gingival mucosa, anterior tonsillar pillar, and inner lips. Five patients displayed ulcerative lesions in the lower lip and vesiculobullous and ulcerative lesions on the tongue and jugal mucosa. Two patients underwent immunohistochemical staining, which indicated the presence of herpes simplex virus (HSV-1). One patient underwent a histopathological analysis that concluded the existence of *Sarcina ventriculi* colonies. The authors concluded that these oral findings are typical for immunocompromised patients and are secondary lesions related to traumatic events or co-infections. Most of these examined patients were hospitalized due to severe SARS-CoV-2 infection and received mechanical ventilation and, as a consequence, developed oral injuries.

Based on the included analytical studies, alterations in taste sensations, xerostomia, and aphthous-like ulcers were the most prevalent oral manifestation in COVID-19 patients. The most common site for these manifestations is the dorsum of the tongue, in which it is believed to be due to epithelial alterations caused by SARS-CoV-2.

### 4.3. Study Limitations

The main limitations of our systematic review should be considered to be the heterogeneity of the included studies. Sources of heterogeneity include individual factors, e.g., age group, gender, race and comorbidities, as well as environmental factors such as hospitalization, treatment implemented or pandemic period. Furthermore, the variety of selected study design and the methods for the evaluation of determined oral manifestations makes it difficult to compare the meta-analysis results with the individual studies. Unfortunately, the studies did not have adequate timeframes for long-term observation of the evolution of changes in the oral mucosa with the development of SARS-CoV-2 infection or improvement of health condition after the hospitalization caused by COVID-19. However, it must be emphasized that the subject is very topical, and at the same time dynamic.

## 5. Conclusions

Our systematic review suggests higher prevalence of oral manifestations in SARS-CoV-2 positive patients, especially xerostomia, ulcerations and taste alterations. However, the relationship between oral health status and SARS-CoV-2 infection cannot be clearly defined due to confounders such as individual or environmental factors.

## Figures and Tables

**Table 1:** Inclusion and exclusion criteria according to the PICOS.

Parameter	Inclusion Criteria	Exclusion Criteria
Population	SARS-CoV-2 positive patients—aged from 0 to 99 years, both genders	patients with other infectious diseases
Intervention	not applicable	
Comparison	not applicable	
Outcomes	determined the presence of oral manifestations, including oral lesions	determined only the presence of gastrointestinal manifestations, such as dysphagia
Study design	case-control, cohort and cross-sectional studies, case series with min. 10 patients	literature reviews, case reports, expert opinion, conference reports
	published between 2020 and 2022	not published in English

**Table 2:** General characteristics of included studies.

Author, Year, Setting	Study Design	Pandemic Period	Participants (F/M); Age [Years]	Comorbidities	Hospitalization and COVID-19 Treatment	Inclusion Criteria	Exclusion Criteria	General Manifestations with Frequency [%]	Smoking Status
Abubakr et al., 2021, Egypt [29]	questionnaire study	1 May to 1 July 2020	573 (408/165); 36.19 ± 9.11 (range: 19–50)	none	non-hospitalized; NR	Egyptian adults, 18–50 years old; laboratory-confirmed COVID-19 infection (PCR test); non-smokers; non-alcoholics; medically free; mild-to-moderate symptoms; good oral hygiene and not suffering from any oral manifestations before the pandemic	failure to complete the whole questionnaire; poor oral hygiene or any of the oral symptoms before the pandemic; chronic illnesses; smokers; alcoholics; serious COVID-19 infection, severe respiratory failure or required hospitalization	muscle pain (76.4), malaise (72.8), headache (70.0), fever (66.0), loss of smell (61.8), cough (55.5), sore throat (52.4), dyspnea (51.8), diarrhea (50.3)	non-smokers
Bardellini et al., 2021, Italy [30]	retrospective cross-sectional study	March to April 2020	27 (8/19); 4.2 ± 1.7 (range: 3 months–14 years)	NR	hospitalized; NR	pediatric patients (aged 0–14 years old), laboratory evidence of COVID-19 infection, signed informed consent	NR	fever > 38 °C (55.5), mild febrile conditions (37.0), cough (37.0), rhinorrhea (25.9), difficulty in breathing (18.5)	NA
Biadsee et al., 2020, Israel [31]	questionnaire study	25 March to 15 April 2020	128 (70/58); 36.25 (range: 18–73)	hypertension (*n* = 8), hypothyroidism (*n* = 4), diabetes mellitus (*n* = 3), asthma (*n* = 2)	non-hospitalized; NR	diagnosed by RT-PCR and considered to have mild symptoms, according to the latest WHO joint report	questionnaires with missing information	cough (59.4), weakness (47.7), myalgia (46.9), fever (42.2), headache (40.6), impaired sense of smell (38.3), sore throat (26.6), runny nose (26.6), nasal congestion (22.7), gastrointestinal symptoms (18.8)	smokers (*n* = 26)
Bulut et al., 2021, Turkey [32]	questionnaire study	September 2020 to March 2021	200 (125/75); ranges: 20–30: 89 (62/27), 31–40: 65 (43/22), 41–50: 27 (14/13), 51–60: 15 (4/11), 61–70: 4 (2/2)	NR	hospitalized (11.5); antiviral drugs (61.7), anticoagulant (16.9), hydroxychloroquine (12.3), antiaggregant (7.1), dexamethasone (5.2), antibiotics (5.2); not use any medication (27.9)	reported COVID-19 in anamnesis	age < 18 years	presence of symptoms (87.5)	NR
Eduardo et al., 2022, Brazil [33]	retrospective cohort study	May 2020 to February 2021	472 (150/322);majority range: 51–80	NR	hospitalized (ICU); orotracheal intubation (89.8), tracheostomy (5.1), nasal catheter (3.8)	adults of both genders, adequate information of oral cavity conditions during ICU hospitalization	oral medicine records without adequate information about gender, age, presence and type of mechanical ventilation, and oral conditions or information about	NR	NR
El Kady et al., 2021, Egypt [34]	pilot questionnaire study	15 May to 10 June 2020	58 (27/31); range: 18–46	NR	hospitalized; NR	adults with SARS-CoV-2 infection confirmed by RT-PCR, and isolated in hospitals	NR	NR	NR
Elamrousy et al., 2021, Egypt [35]	cross-sectional study	2 September 2020, to 10 June 2021	124 (32/92); 50.32 ± 12.47	diabetes (*n* = 52), hypertension (*n* = 16), cardiac disease (*n* = 8), renal disease (*n* = 4), liver disease (*n* = 4)	hospitalised; zithrocin (100.0), iverzine (100.0), zinc, vitamin C (100.0), anticoagulant (90.3), antibacterial (70.9), prednisolone (61.3), remdesivir (22.6), acetylcysteine (19.3), foradil (12.9), colchicine or hydroxychloroquine (12.9), antihypertensive (12.9), silymarin (3.2)	adults with SARS-CoV-2 infection confirmed by RT-PCR	without a laboratory-confirmed diagnosis of COVID-19 infection, olfactory or gustatory impairment prior to COVID-19 infection, malignant neoplasms or neurodegenerative diseases	asthenia (67.7), breath problems (67.7), cough (67.7), fatigue (19.4), abdominal symptoms (12.9)	NR
Fantozzi et al., 2020, Italy [36]	questionnaire study	6 March to 30 April 2020	111 (53/58); median 57 (range: 48–67)	hypertension (*n* = 29), chronic pulmonary disease (*n* = 11), diabetes (*n* = 10), cardiovascular disease (*n* = 9), cancer (*n* = 5)	hospitalized; NR	adults with confirmed SARS-CoV-2 infection	NR	fever (90.9), cough (46.8), dyspnea (34.3), diarrhea (4.5), sore throat (3.6), fatigue (3.6), myalgia/arthralgia (2.7), vomiting (2.7)	smokers (*n* = 7), former smokers (*n* = 38)
Favia et al., 2021, Italy [37]	observational study	October to December 2020	123 (53/70); median 72	NR	hospitalized; NR	adults with SARS-CoV-2 infection confirmed by RT-PCR after nasal and oropharyngeal swabs	certain pre-existing lesions, symptomatic of pre-existing systemic and local conditions previously diagnosed and well-known to the patients, as well as traumatic lesions; the asymptomatic and mild forms	fever, anosmia, cough, sore throat, congestion and runny nose, nausea or vomiting, muscle and body aches, dermatologic manifestation, pneumonia, dyspnea and hypoxia, acute respiratory distress syndrome, multi-organ failure	NR
Fidan et al., 2021, Turkey [38]	prospective observational study	April to October 2020	74 (25/49); 51.4 ± 6.3 (range: 28–68)	NR	hospitalized; NR	infection confirmed by RT-PCR of nasopharyngeal swab	hormone therapy and/or steroid therapy in the one month prior to the study or taking any drugs that might affect oral lesion; oral lesions prior COVID-19 diagnosis	NR	NR
Ganesan et al., 2022, India [39]	observational cross-sectional study	18 October to 7 November 2020	500 (133/367); 53.46 ± 17.50	NR	hospitalized; NR	hospitalized, age ≥ 16 years, treated in the institute	pediatric population, any other systemic conditions affecting oral mucosa	influenza-like illness (64.6), severe acute respiratory infection (18.4)	smokers (*n* = 166)
Gherlone et al., 202, Italy [40]	retrospective and prospective cohort study	23 July to 7 September 2020	122 (30/92); median 62.5 (IQR 53.9–74.1)	hypertension (*n* = 50), diabetes mellitus (*n* = 17), coronary artery disease (*n* = 12), chronic kidney disease (*n* = 9), chronic bronchopulmonary disease (*n* = 8), active neoplasia (*n* = 7)	hospitalized survivors; antibiotics (83.6), biologics (31.1), steroids (29.5)	adults admitted to the emergency department; positive SARS-CoV-2 nasopharyngeal swab on RT-PCR in the presence of clinical and/or radiological signs of COVID-19; signed the informed consent	NR	NR	smokers (*n* = 48)
Halepas et al., 2021, USA [41]	retrospective cross-sectional study	15 March to 1 June 2020	47 (23/24); 9.0 ± 5.0 (range: 1.3–20.0)	NR	hospitalized; NR	21 years or younger, fever of prolonged duration, laboratory evidence of inflammation, required hospitalization, multiorgan involvement, confirmed positive COVID RT-PCR or serology test results	NR	multisystem inflammatory syndrome in children; fever > 5 days (100.0), systemic rash (68.1), conjunctivitis (57.5), vomiting (51.1), diarrhea (38.3), myocarditis (36.2), cervical lymphadenopathy (19.2), cough (14.9), irritability (14.9), cranial nerve palsy (12.8), pericardial effusion (12.8), extremity edema (12.8), arthritis (8.5), rhinorrhea (6.4)	NR
Katz and Yue, 2021, USA [42]	registry study	NR	895 (386/509); 0–9: 5.33%, 10–17: 2.47, 18–34: 39.9, 35–44: 11.1, 45–54: 1.2, 55–64: 11.2, 65–74: 12.3, 74–85: 16.5	respiratory disease, endocrine disease, obesity, diabetes, circulatory disease	hospitalized; NR	diagnosis of COVID-19 ICD-10-U07.1 and/or ICD-10-K12.0 (recurrent aphthous stomatitis)	none	NR	smokers and non-smokers (NR)
Naser et al., 2021, Iraq [43]	prospective study	August 2020 to March 2021	338 (138/200); mean 42.1	hypertension (*n* = 235), diabetes mellitus (*n* = 218), heart diseases (*n* = 108), renal diseases (*n* = 69), blood diseases (*n* = 37), respiratory disorders (*n* = 32), gastrointestinal diseases (*n* = 27), liver diseases (*n* = 26)	hospitalized; NR	diagnosed with COVID-19 + by PCR; age ≥ 10 years, with acute oral or perioral lesions either during admission or which appeared later during treatment; nonsmoker, no alcohol consumption	age ≤ 10 years; lesions appeared or were well established before SARSCoV-2 infection; not tolerating follow-up or refusing to enroll in study	NR	non-smokers
Natto et al., 2021, Saudi Arabia [44]	pilot cross-sectional study	28 July 28 to 5 October 2020	109 (36/73); 39.3 ± 12.4	diabetes (*n* = 11), hypertension (*n* = 8), asthma (*n* = 3), epilepsy and arthritis (*n* = 2)	non-hospitalized; NR	symptomatic and non-hospitalized, diagnosed with COVID-19 through a nasopharyngeal swab using RT-PCR	age < 18 years or suspected patients without any definitive diagnosis	muscle pain (77.1), fever (67.9), cough (50.5), headaches (49.5), no smell (44.0), sore throat (35.8), diarrhea (31.2), shortness of breath (22.0), nausea (20.2), runny nose (15.6), vomiting (9.2)	non-smokers (majority)
Nuno-Gonzalez et al., 2021, Spain [45]	cross-sectional study	10 to 25 April 2020	666 (386/280); mean 55.7	NR	hospitalized; NR	positive RT-PCR testing for SARS-CoV-2, or bilateral pneumonia; only adults; mild-to-moderate COVID-19 pneumonia	NR	NR	NR
Rafałowicz et al., 2021, Poland [46]	observational study	until mid-2021	1256	present (NR)	survivors—hospitalized (about 30.0); NR	infected with SARS-CoV-2 in the period from 2 to 6 months before the visit	NR	fever, malaise, anosmia, pneumonia, diarrhea, vomiting, fatigue, irritability, trouble sleeping and concentrating, sweating, amnesia, shortness of breath, palpitations	NR
Riad et al., 2020, Egypt [47]	case series	April to August 2020	13 (8/5); 51.08 ± 8.79 (range: 34–62)	diabetes (*n* = 3), hypertension (*n* = 2), asthma (*n* = 2)	non-hospitalized; dexamethasone (*n* = 2), chloroquine (*n* = 2), paracetamol (*n* = 9)	generalized pain and soreness within the oral cavity related mainly to non-keratinized mucosa without a specific cause; positive RT-PCR testing for SARS-CoV-2	NR	ageusia (30.8), fever (15.4), anosmia (15.4)	smokers (*n* = 3)
Riad et al., 2021, Egypt [48]	case series	May to August 2020	18 (14/4); 35.11 ± 13.3 (range: 18–72)	NR	non-hospitalized; paracetamol (*n* = 4), ibuprofen (*n* = 3), prednisolone (*n* = 1), chloroquine (*n* = 1)	offensive oral malodor that precipitated notable psychosocial distress; positive RT-PCR testing for SARS-CoV-2	NR	fever (11.1), anosmia (11.1), dry cough (5.6), ageusia (5.6)	smokers (*n* = 3)
Riad et al., 2022, Egypt [49]	case series	April to June 2020	26 (17/9); 36.81 ± 15.65 (range: 16–70)	none	non-hospitalized; paracetamol	pain in the tongue; positive RT-PCR testing for SARS-CoV-2	NR	fever (15.4), ageusia (11.5), sore throat (7.7), dry cough (3.8)	NR
Said Ahmed et al., 2021, Egypt [50]	case series	NR	14 (4/10); mean 54.2	diabetes	hospitalized (71.4); NR	14–30 days post COVID-19 recovery, complaining from pain, loss of one or more maxillary teeth, exposed bone, pus discharge, and bad odor	NR	NR	NR
Sinjari et al., 2020, Italy [51]	observational study	May to June 2020	20 (9/11); mean 69.2	hypertension (*n* = 8), thyroid disorders (*n* = 5), diabetes (*n* = 3), obesity (*n* = 3)	hospitalized; lopinavir/ritonavir and/or hydroxychloroquine	both gender and of any age, hospitalized for COVID-19, able to give consent to participate in the study	in need of intensive care and/or unable to give consent to participate in the study or unable to intend or to want	NR	smokers (*n* = 2)
Soares et al., 2022, Brazil [52]	case series	NR	14 (4/10); mean 58 (range: 23–88)	hypertension (*n* = 4), diabetes (*n* = 3), chronic obstructive pulmonary disease (*n* = 1)	non-hospitalized; NR	positive RT-PCR testing for SARS-CoV-2; oral lesions	NR	Dysgeusia, anosmia, fever, headache, dyspnea, dry cough	NR
Subramaniam et al., 2021, India [53]	short-term observational study	April to June 2020	713 (297/416); range: 12–80	diabetes, hypertension, asthma	hospitalized; multivitamins and vitamin C following prescribed treatment norms for the care of COVID-positive patients	both genders infected with SARS-CoV-2, diagnosed on RT-PCR in the age group of 12–80 years, admitted to the hospital	age < 12 and >80, not willing to give written informed consent, seriously ill requiring intensive care	complaints ranging from mild fever, sore throat, to difficulty in breathing	NR
Villarroel-Dorrego et al., 2022, Venezuela [54]	observational study	NR	55 (25/30); 51 ± 23.24 (range: 1–89)	hypertension (*n* = 10), asthma (*n* = 9)	hospitalized (ICU: 34.5); combination of lopinavir and ritonavir, dexamethasone and remdesivir (ICU)	hospitalized with COVID-19 confirmed by PCR and a rapid antigen FIA diagnostic test	NR	NR	NR
Zarpellon et al., 2021, Brazil [55]	case series	NR	26 (15/11); mean 50 (range: 8–76)	hypertension or diabetes	hospitalized (ICU); mechanical ventilation support	SARS-CoV-2 positive deceased who were admitted in ICU	NR	severe acute respiratory syndrome	smokers (*n* = 2)

Legend: F, females; M, males; NR, not reported; ICU, intensive care unit; RT-PCR, reverse transcription polymerase chain reaction; FIA, fluorescent immunoassay.

**Table 3:** Detailed characteristics of included studies considering oral manifestations.

Author, Year	Investigation	Prevalence of Oral Manifestations [%]	Type of Oral Manifestations with Location and Frequency [%]
Abubakr et al., 2021 [29]	self-report survey	71.7	loss of taste (55.5), xerostomia (47.6), oral or dental pain (23.0), ulcerations (20.4), pain in jaw bones or joint (12.0), halitosis (10.5); 2 or 3 manifestations simultaneously (28.3)
Bardellini et al., 2021 [30]	physical examination	55.6	taste alteration (11.1), oral pseudomembranous candidiasis (7.4), geographic tongue (3.7), coated tongue (7.4) and hyperemic pharynx (37.0)
Biadsee et al., 2020 [31]	self-report survey	NR	xerostomia (56.3), changes in taste sensation (52.3), masticatory muscle pain (11.7), change in sensation in the tongue (9.4), swelling in the oral cavity (7.8): 4 in the palate, 4 in the tongue and 2 in the gums, plaque-like changes in the tongue (7.0), oral bleeding (4.7)
Bulut et al., 2021 [32]	self-report survey	NR	taste loss (53.0), halitosis (21.0), oropharyngeal wound and pain (18.0), pain in the chewing muscles (16.0), pain in the temporomandibular joint (17.5), gum bleeding (17.5), xerostomia (38.0, after recovery 12.0), aphthous ulcer (14.5), sensitivity and/or pain in teeth (12.0), herpes labialis (8.5), burning in the tongue (7.5)
Eduardo et al., 2022 [33]	physical examination	51.3	mechanical trauma (18.1), viral infection (11.4), unspecific erosive lesions (10.5), petechial/hematoma (10.5), dryness of oral mucosa (9.9), oral bleeding (7.5), dryness of lips (6.0), dental associated lesions (6.0), candidiasis (5.4), edema (3.6), sialorrhea (3.3), tongue coating (3.0), varicose (2.4); 2 or 3 concomitant alterations (23.2)
El Kady et al., 2021 [34]	self-report survey	67.2	dry mouth (39.7), gustatory dysfunction as loss of salt sensation (34.5), loss of sweet sensation (29.3), and altered food taste (25.9), burning sensation in mouth or tongue (22.4), difficult swallowing (22.4), oral ulcers (17.2), spots of mouth or lips (13.8), pain or swelling in the salivary gland or cheek (13.8), pain or swelling below the mandible (10.3), tongue redness (8.8), gingival bleeding (7.0)
Elamrousy et al., 2021 [35]	physical examination	90.3	dry mouth (83.9), ulcers (81.3), taste loss (54.8), candidiasis (37.5), hyperpigmentation (22.6), atrophic tongue (15.6), petechiae (15.6), burning sensation (14.3), tongue coating (9.4), herpes infection (6.3), white lesions (6.3); tongue (75.0), labial mucosa (59.4), buccal mucosa (46.9), lips (40.6) floor of mouth (34.4) and gingiva (12.5)
Fantozzi et al., 2020 [36]	survey	NR	taste dysfunction (59.5), xerostomia (45.9), swallowing difficulties (18.0)
Favia et al., 2021 [37]	physical examination	65.9	ulcerative lesions (52.8), hyperplasia of papillae (39.0), candidiasis (22.7), blisters (15.4), petechial (11.4), angina bullosa (8.9), ulcero-necrotic gingivitis (5.6), geographic tongue (5.6) and fissured tongue (4.0)
Fidan et al., 2021 [38]	physical examination	78.4	aphthous-like ulcer (36.5), erythema (25.7), lichen planus (16.2); tongue (31.8), buccal mucosa (27.0), gingiva (14.9), palate (5.4)
Ganesan et al., 2022 [39]	physical examination	NR	alteration in taste sensation (51.2), xerostomia (28.0), erythematous macules with burning sensation (7.2), atrophic glossitis (4.6), non-specific solitary ulcers (3.0), candida-like lesions (1.0), white patch (1.0), ductal inflammation (0.4)
Gherlone et al., 2021 [40]	physical examination	83.9	salivary gland ectasia (43.0), white tongue (29.0), dry mouth (24.0), masticatory muscle weakness (19.0), oral ulcers (11.0), temporomandibular joint abnormalities (7.0)
Halepas et al., 2021 [41]	physical examination	55.3	red or swollen lips (48.9), strawberry tongue (10.6)
Katz and Yue, 2021 [42]	physical examination	NR	recurrent aphthous stomatitis (0.67; OR = 14.0)
Naser et al., 2021 [43]	physical examination	NR	loss of taste (79.5), white coats of the tongue (31.6), pain related to the oral cavity (27.8), multiple aphthous ulceration (24.8), dryness (24.5), dysphagia (23.0), white coats of cheeks and gingiva (22.4), white coats of the palate (15.6), single aphthous ulceration (9.1)
Natto et al., 2021 [44]	physical examination	53.2	loss of taste (43.4), erythema/desquamated gingivitis (7.3), coated tongue (7.3), ulcers/blisters (6.4)
Nuno-Gonzalez et al., 2021 [45]	physical examination	11.7	anterior U-shaped lingual papillitis (5.3), aphthous stomatitis (3.2), tongue swelling (3.0), burning sensation in the mouth (2.4), mucositis (1.8), glossitis with patchy depapillation (1.8), candidiasis (0.5), enanthema (0.3)
Rafałowicz et al., 2021 [46]	physical examination	NR	discoloration, ulceration, and hemorrhagic changes on the oral mucosa (32.0), mycosis located on the tongue (29.7), unilateral (more often left-sided) aphthous-like lesions on the hard palate (25.8), atrophic cheilitis (12.5); approx. 60% salivary secretory disorders in the initial period of infection, which in 6.68% prolonged up to 4 months after systemic symptoms disappeared
Riad et al., 2020 [47]	physical examination	NA	mucositis (intraoral pain, sporadic erythema with minor irritations); all over the mouth (53.8), on the buccal mucosa (30.8), palate (15.4), and gingiva (7.7)
Riad et al., 2021 [48]	physical examination	NA	halitosis (the majority with ‘fair’ level of oral hygiene, except for two patients (11.1) with a ‘poor’ level, while one patient (5.5) further complicated by an intraoral ulcer)
Riad et al., 2022 [49]	physical examination	NA	tongue ulcers (92.3 of them not bleeding, ranged between 1 and 7 ulcers per patient, and their size ranged between 1 and 5 mm corresponding to herpes-like ulcers with scalloped borders); all of them manifested on dorsum or side of the tongue, while 4 (15.4) on the ventral surface
Said Ahmed et al., 2021 [50]	physical examination	NA	maxillary mucormycosis osteomyelitis
Sinjari et al., 2020 [51]	self-report survey	NR	xerostomia (30.0), impaired taste (25.0), difficulty in swallowing (20.0, burning sensation (15.0)
Soares et al., 2022 [52]	physical examination	100.0	petechial, ecchymosis, reddish macules, and chronic ulcers with more than 7 d of evolution, vesiculobullous eruptions; only the palate (57.1), tongue (28.6), either the lip or palate (14.3)
Subramaniam et al., 2021 [53]	physical examination	1.26	ulcers on the buccal mucosa, burning sensation, generalized mucositis on the labial mucosa, erythema of tongue margins, tongue papillary atrophy, bilateral angular cheilitis, reddish-white spots on the palate
Villarroel-Dorrego et al., 2022 [54]	physical examination	40.0	alteration or a total loss of taste (60.0), pain or burning in the mouth (36.4), xerostomia (27.3), candidiasis (12.7), hemorrhagic ulcerative lesions (7.3), multiple ulcerations resembling cancer sores (5.5), lingual varicose veins (5.5), migratory glossitis (5.5), enanthems in the labial or cheek mucosa (3.6), severe angular cheilitis (1.8), white plaques (1.8) and lichenoid lesions (1.8), recurrent cold sore (1.8)
Zarpellon et al., 2021 [55]	physical examination (post mortem)	NA	vesiculo-bullous and ulcerative lesions in the oral mucosa, such as tongue, lips and buccal mucosa

Legend: NR, not reported; NA, not applicable.

## Data Availability

Data are available on request from the corresponding author.
